# Structural characterization of the C-terminal domain of SARS-CoV-2 nucleocapsid protein

**DOI:** 10.1186/s43556-020-00001-4

**Published:** 2020-08-06

**Authors:** Renjie Zhou, Rui Zeng, Albrecht von Brunn, Jian Lei

**Affiliations:** 1 National Clinical Research Center for Geriatrics, State Key Laboratory of Biotherapy and Cancer Center, West China Hospital, Sichuan University, No.17, Block 3, Southern Renmin Road, Chengdu, 610041 Sichuan China; 2 Max von Pettenkofer-Institute, Ludwig-Maximilians-University Munich and German Center for Infection Research (DZIF), Partner Site Munich, 80336 Munich, Germany

**Keywords:** Coronavirus, SARS, Nucleocapsid, Viral RNA, Structure

## Abstract

The newly emerging severe acute respiratory syndrome coronavirus 2 (SARS-CoV-2) has resulted in a global human health crisis. The CoV nucleocapsid (N) protein plays essential roles both in the viral genomic RNA packaging and the regulation of host cellular machinery. Here, to contribute to the structural information of the N protein, we describe the 2.0 Å crystal structure of the SARS-CoV-2 N protein C-terminal domain (N-CTD). The structure indicates an extensive interaction dimer in a domain-swapped manner. The interface of this dimer was first thoroughly illustrated. Also, the SARS-CoV-2 N-CTD dimerization form was verified in solution using size-exclusion chromatography. Based on the structural comparison of the N-CTDs from *alpha-*, *beta-*, and *gamma-*CoVs, we demonstrate the common and specific characteristics of the SARS-CoV-2 N-CTD. Furthermore, we provide evidence that the SARS-CoV-2 N-CTD possesses the binding ability to single-stranded RNA, single-stranded DNA as well as double-stranded DNA in vitro. In conclusion, this study could potentially accelerate research to understand the complete biological functions of the new CoV N protein.

## Introduction

Over the past two decades, the emergence of novel human coronaviruses has caused continuous threats to the public health systems worldwide. Severe acute respiratory syndrome coronavirus (SARS-CoV) infected more than 8000 people with ~ 10% fatality rate during the global outbreak of 2002/2003 [1]. Ten years later, another highly pathogenic agent, the Middle-East respiratory syndrome coronavirus (MERS-CoV) appeared in the Arabian Peninsula and currently infected approximately 2500 cases with a ~ 35% mortality rate (www.who.int). At the end of 2019, a new coronavirus (CoV), SARS-CoV-2 (also named 2019-nCoV) was identified (GenBank: MN908947, [2]). This new CoV could cause the so-called coronavirus disease 2019 (COVID-19). COVID-19 includes various symptoms from the general mild respiratory disease (i.e. sneezing and coughing) to severe pneumonia as well as enteric disease [3]. As of July 08, 2020, more than 11.5 million confirmed SARS-CoV-2 infection cases have been reported globally, with more than 535,000 deaths (https://covid19.who.int/). However, no efficient vaccine or treatment is available so far.

CoVs belong to the family *Coronaviridae* of the order *Nidovirales* and infect various species [4], such as human, cattle, pigs, cats, and birds *etc*. They are enveloped positive-sense single-stranded (+ss) RNA viruses with the largest genome of all currently known RNA viruses. CoVs are divided into four genera: *alpha-*, *beta-*, *gamma-*, and *delta-coronaviruses* [5]. SARS-CoV-2, similar to SARS-CoV and MERS-CoV, belongs to the *beta-coronavirus* group. Its genome comprises about 29.9 kb nucleotides and shares ~ 80% of genome identity with SARS-CoV [6]. The 5′-terminal two-thirds of its genome contain ORF1a and ORF1a/b encoding sixteen nonstructural proteins Nsp1–16. They are mainly responsible for the membrane-associated viral replication/transcription complex (RTC) formation [7]. The 3′-proximal one-third encodes four structural proteins: the spike (S), envelope (E), membrane (M, also called matrix protein) and nucleocapsid (N) proteins, as well as several accessory proteins. The S protein mediates the virus recognition of the host cell surface receptor and subsequent membrane fusion [8]. The E and M proteins are mainly involved in viral envelope formation, virus assembly, and viral particle budding [9, 10]. The N protein is a multi-functional protein and plays many roles in the viral life cycle [11, 12].

As a diagnostic marker [13], the coronavirus N protein is present in a large copy number in the CoV-infected host cells. The primary function of the CoV N protein is to bind the viral RNA and form a helical ribonucleoprotein (RNP) complex, in order to protect the viral genome and maintain reliable viral replication [14–16]. Furthermore, the SARS-CoV N regulates the host innate immune response by inhibiting interferon β (IFN-β) production [17, 18]. However, the exact mechanisms of these processes are still unclear. The CoV N is also involved in binding other viral proteins. It interacts with the E and M proteins to facilitate virus envelope formation and particle assembly [19–21]. MHV (murine hepatitis virus, a *beta*-CoV) N protein binds Nsp3 to tether the viral genome and RTC complex [22, 23]. In addition, the N protein interacts with numerous host cell proteins as well. For example, the SARS-CoV N directly binds to host translation elongation factor 1α (EF1α) to inhibit host cell proliferation, including that of peripheral blood lymphocytes [24]. It also interacts with the complex cyclin-CDK (cyclin dependent kinase) to regulate the cell cycle for facilitating CoV replication [25]. Recently, using affinity-purification mass spectrometry methods to systematically identify all SARS-CoV-2 viral proteins and host protein interactions, the SARS-CoV-2 N was described to potentially interact with 15 human proteins [26]. Most of them are related to RNA processing (i.e. Polyadenylate-binding protein 1 and 4: PABP-1 and PABP-4) or stress granule regulation (e. g., G3BP-1: Ras GTPase-activating protein-binding protein 1, G3BP2). Although these interactions have to be further investigated, they imply the major ability of CoV N protein to interact with other proteins.

**Fig. 1:**
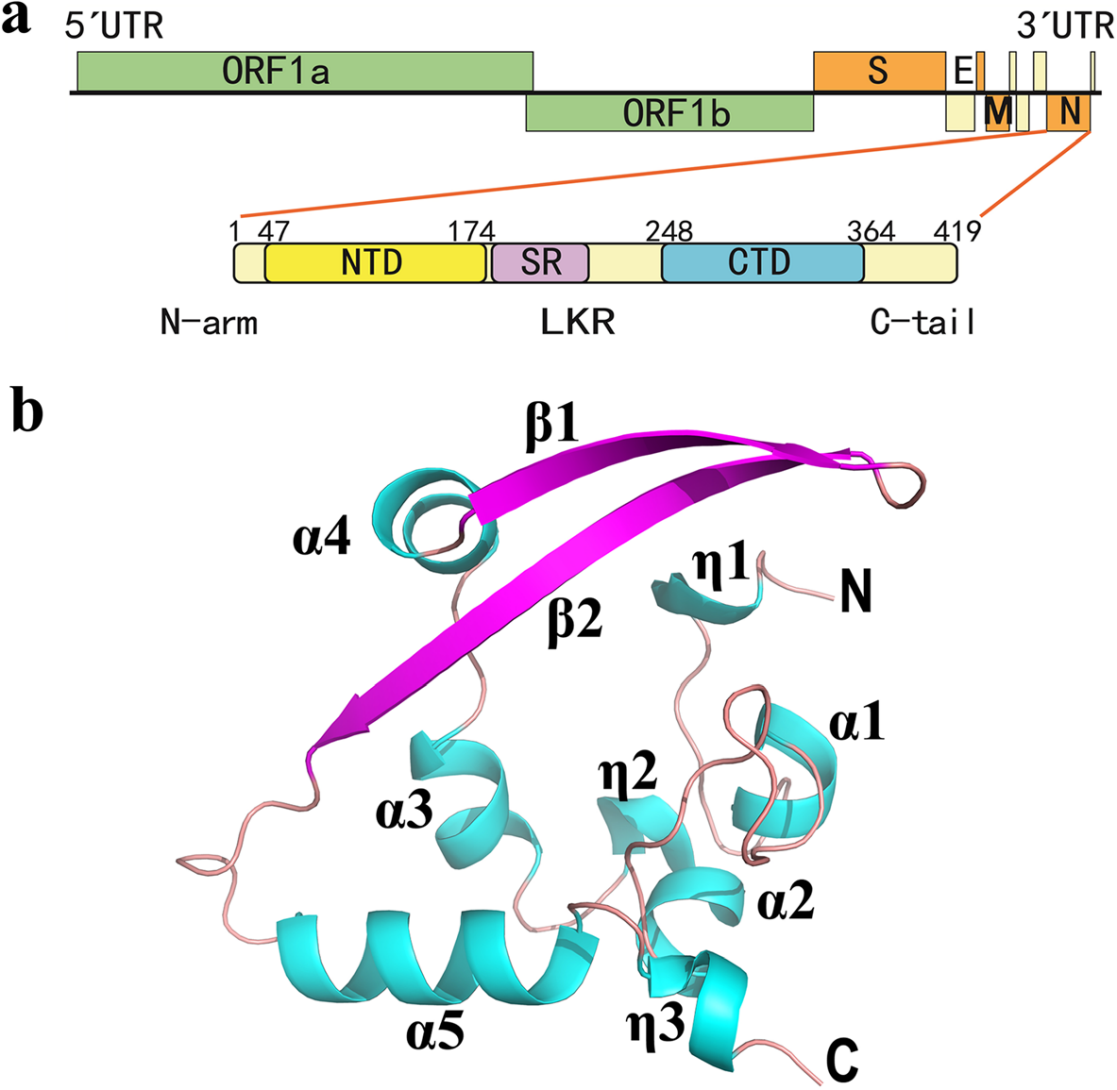
Overall structure of the SARS-CoV-2 N-CTD. **a** Genome organization of coronavirus and the domain composition of the SARS-CoV-2 nucleocapsid (N) protein. ORF: open reading frame; S: spike; E: envelope; M: membrane. N protein consists of the N-terminal arm (N-arm), the N-terminal domain (NTD), the central linker region (LKR) within a Ser/Arg (SR)–rich motif, the C-terminal domain (CTD), and the C-terminal tail (C-tail). **b** Ribbon view of the overall structure of the SARS-CoV-2 N-CTD monomer. The structure of N-CTD contains three 3_10_ (η) helices, five α helices, and two antiparallel β-strands with the order η1-α1-α2-η2-α3-α4-β1-β2-α5-η3. Helices are shown in cyan, strands in purple. The N and C termini of the N-CTD are marked. Fig. **b** was prepared with the program PyMOL (https://pymol.org)

All CoV N proteins possess a conserved modular composition comprising two structured domains, the N-terminal domain (NTD) and the C-terminal domain (CTD), interspersed by three intrinsically disordered regions (IDRs) known as the N-terminal arm (N-arm), the central linker region (LKR) within a Ser/Arg (SR)-rich motif, and the C-terminal tail (C-tail) [11, 27] (Fig. 1a). The N-NTD, N-CTD and C-tail domains all were reported to bind viral RNA in SARS-CoV [28–30]. The SR-rich region in the LKR could regulate the N protein oligomerization upon phosphorylation [31]. The N protein self-association is necessary for viral RNP assembly [32]. The N-CTD has been shown to directly participate in N protein dimerization and oligomerization [29, 30, 32, 33]. Furthermore, the N protein inhibits IFN-β and binds to EF1α (mentioned above) mainly through the N-CTD [18, 24].

To date, the N-CTD structures of SARS-CoV, IBV (infectious bronchitis virus), MHV (mouse hepatitis virus), HCoV-NL63, and MERS-CoV have been determined by NMR [30] and crystallography [29, 33–37]. During preparation of this manuscript, the structure of SARS-CoV-2 N-CTD was reported on the BioRxiv server [38]. These authors intensely discuss the architecture and self-assembly properties of the N protein in SARS-CoV-2, however, the detailed interactions between the N-CTD dimer and the evidence of the N-CTD binding to nucleotides are not reported. In the present study, we determined the crystal structure of SARS-CoV-2 N-CTD at a resolution of 2.0 Å, and we report the interactions of the N-CTD homo-dimer in strong detail. We further identified this dimer in solution. The common and specific characteristics of the SARS-CoV-2 N-CTD are discussed. In addition, we provide direct evidence that the SARS-CoV-2 N-CTD can interact with ssRNA, ssDNA and double-stranded DNA (dsDNA) in vitro. These findings facilitate detailed understanding of the new CoV-2 N protein.

## Results and discussion

### Overall structure of the C-terminal domain of SARS-CoV-2 N protein (N-CTD)

We designed a SARS-CoV-2 (GenBank: MN908947, [2]) N-CTD construct containing the residues from Lys248 to Pro364 of the N protein (Fig. 1a) and determined the three-dimensional structure of the corresponding region at 2.0 Å. The diffraction parameters and refinement statistics are summarized in Table 1. Four molecules (termed as A, B, C and D) formed by two dimers exist in the asymmetric unit (ASU) of the N-CTD crystal. In the final model, residues 256–364 of Chain A, 252–364 of Chain B, 253–364 of Chain C, as well as 257–364 of Chain D were successfully built. The side-chains of the following residues showed the double conformation: Thr329 in Chain A; Thr282, Met317 and 322 in Chain B; and Thr329, 334 in Chain D. The root mean square deviation (RMSD) values between any two subunits of these four molecules are 0.2–0.9 Å for all Cα atoms, as calculated by the DaLi server [40]. Therefore, the overall structures of each molecule are almost identical.

**Table 1:** Data collection and refinement statistics

	SARS-CoV-2 N-CTD PDB code: 7C22
***Data collection statistics***	
Space group	*P*_*1*_
Unit-cell dimensions (Å, °)	*a* = 43.76, *b* = 49.46, *c* = 68.82
	*α* = 106.79, *β* = 90.04, *γ* = 97.79
Wavelength (Å)	0.97851
*V*_m_ (Å^3^/Da)	2.69
Solvent content (%)	52.24
Resolution range (Å)	19.81–2.00 (2.05–2.00)
Number of unique reflections	35,328 (2636)
R_merge_	0.107 (0.639)
Completeness (%)	95.6 (96.0)
Mean I/σ(I)	5.9 (1.4)
Multiplicity	3.5 (3.5)
CC_1/2_	0.995 (0.752)
***Refinement statistics***	
*R*_*factor*_ (%) ^a^	18.9
*R*_*free*_ (%)^a^	23.8
*No. of atoms*	
Protein	3568
Ligand	15
Water	395
Clashscore^b^	7
r.m.s.deviation in bond lengths (Å)	0.007
r.m.s.deviation in bond angles (°)	1.437
Average *B*-factor for all atoms (Å^2^)	26.0
*Ramachandran plot*	
Residues in preferred regions (%)	96.24
Residues in allowed regions (%)	3.76
Residues in outlier regions (%)	0.00

^a^*R*_*factor*_ = ∑_hkl_│F_o_(hkl)-F_c_(hkl)│/ ∑_hkl_ F_o_(hkl). *R*_*free*_ was calculated for a test set of reflections (4.9%) omitted from the refinement

^b^Clashscore is defined as the number of clashes calculated for the model per 1000 atoms (including hydrogens) of the model. Hydrogens were added by *MolProbity* [39]

**Fig. 2:**
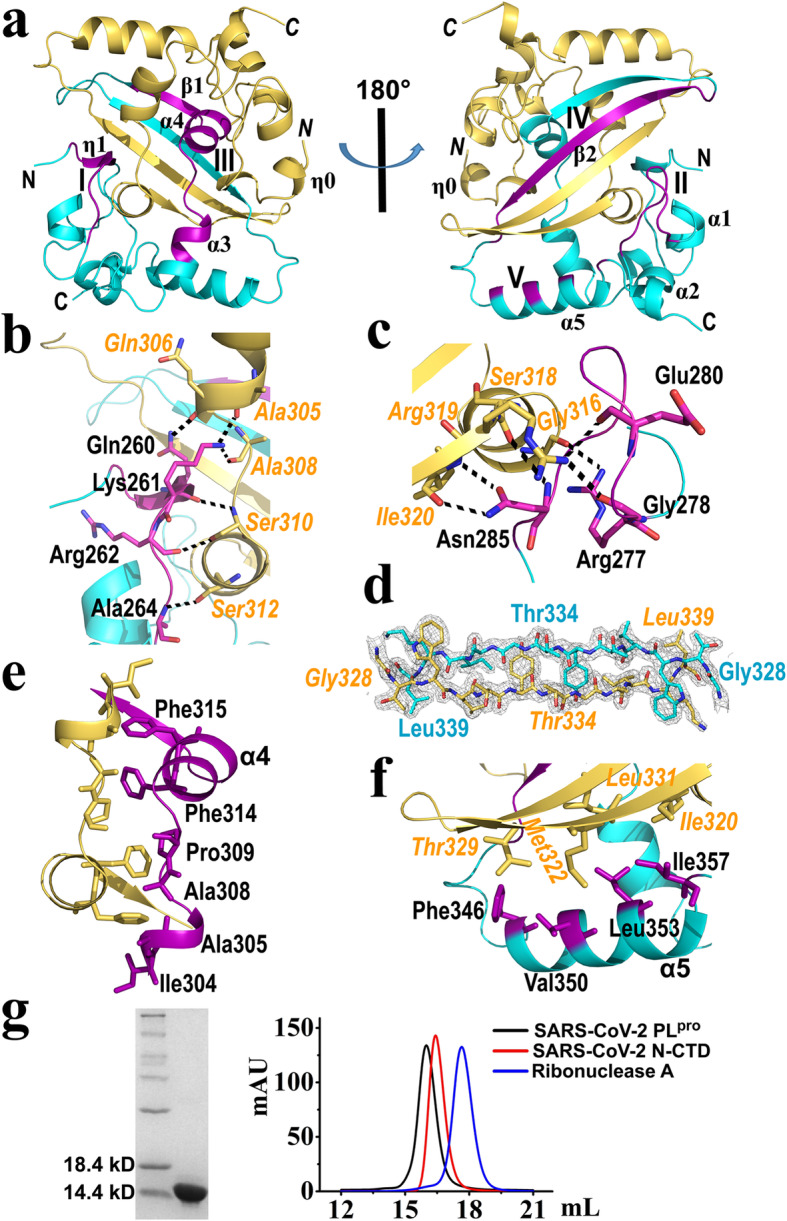
Dimerization of the SARS-CoV-2 N-CTD. **a** Ribbon diagram of the N-CTD dimer. The overall structure of the dimer displays a rectangular slab with all helices at one side (left image) and the β-sheet at the opposite side (right image). Monomers A and B are depicted in cyan and yellow, respectively. The regions **I** and **III** for the N-CTD dimerization are marked in the left image while the regions **II**, **IV** and **V** are indicated in the right one. N- and C- termini of subunit A and B (in italics) are labelled. **b** The hydrogen-bond network in region **I** for the N-CTD dimerization. Residues in protomer A are marked in black, while residues from subunit B are labelled in italics in orange. **c** The hydrogen-bond network in region **II** for the N-CTD dimerization. **d** The 2Fo-Fc electron density (gray, 1.5σ) of the β2 strands (residue Gly328-Leu339; region **IV**) of both subunits. Residues (N- and C- termini) of the β2 are labelled. The non-conserved residue Thr334 is also marked. **e** and **f** The conserved hydrophobic cores in region **III and V** for the N-CTD dimerization. **g** Analytical gel filtration assay was performed. The molecular mass of the SARS-CoV-2 N-CTD is ~ 13.4 kD, which is identified by the SDS-PAGE (left image). In the gel filtration assay, the N-CTD is shown as a dimer in solution (right image). Ribonuclease A (blue line; Sangon Biotech, China): ~ 13.7 kD. SARS-CoV-2 papain-like protease (PL^pro^; black line): ~ 35.5 kD. The peak position of N-CTD (red line) is corresponding to ~ 29.8 kD, representing a dimer of N-CTD (theoretical molecular mass is ~ 26.7 kD). Figures **a-f** were made by the program PyMOL (https://pymol.org)

According to the DSSP (assigning secondary structure of protein) server [41], the structure of N-CTD contains three 3_10_ (η) helices, five α helices, and two antiparallel β-strands (forming a β-hairpin) in all four subunits, with the order η1-α1-α2-η2-α3-α4-β1-β2-α5-η3 (Fig. 1b). The topology shape of N-CTD monomer resembles the letter “C”, forming by the β-hairpin and extending to the rest of the molecule. Interestingly, the structure of subunit B contains one extra 3_10_ helix (named η0) formed by four residues Glu253-Lys256 (Fig. 2a). These residues are partially or completely missing in molecules A and D due to the lack of electron density. Subunit C includes Glu253-Lys256 with poor electron density but these four residues do not form a 3_10_ helix. These results indicate that the N-termini of the N-CTD are somehow flexible. Takeda *et al*. reported the N-terminal residues 248–259 of the SARS-CoV N-CTD (corresponding to residues 247–258 in SARS-CoV-2), which are disordered in NMR structure but more rigid in the crystal structure [30]. These authors suspected that the differences were likely due to crystal packing [30]. However, the N-terminal region of N-CTD is indeed involved in its dimerization/oligomerization (see below). Therefore, future research should investigate whether these conformational changes in the N-termini region could be related to the N protein associations in vivo.

The monomeric N-CTD shows a loose and, − to a certain extent−, an extended conformation with a huge cavity in the center of its structure (Fig. 1b). Thus, the single-molecule conformation is most likely unstable. Dimerization (or oligomerization) is necessary to create a compact and stable conformation for this protein.

### Dimerization of the SARS-CoV-2 N-CTD

Dimerization of N-CTD (molecules A/B, or C/D) exists in our crystal structure. The overall shape of the dimer (here using the A/B dimer as an example) presents a rectangular slab with the two β-hairpins at one side and all helices at the opposite side (Fig. 2a). Approximately 2600 Å^2^ of the surface from each N-CTD monomer (A and B) are buried upon dimer formation, as calculated by the PDBePISA server [42]. The predominant dimerization interface of each subunit includes two β strands and η1, α3, α4 and α5 helices, located in five regions from N-terminus to C-terminus of the N-CTD (Fig. 2a): **I**, residues Pro258-Ala264 (η1 plus the partial loop between η1 and α1); **II**, residues Arg277-Phe286 (the middle region of the loop between α1 and α2); **III**, residues Ile304-Met322 (almost the whole region α3-α4-β1); **IV**, residues Gly328-Leu339 (β2 strand); **V**, residues Phe346-Ile357 (α5 helix).

In regions **I, II** and **IV**, the interactions between molecules A and B mainly refer to hydrogen bonding. In region **I**, residues Gln260-Ala264 of subunit A form six hydrogen bonds with *Ala305-Ser312* (the residues from subunit B are in italics and as follows) of protomer B (Fig. 2b), and vice versa. Notably, the side-chain of the conserved residue Lys261 is involved in two hydrogen bonds with the main-chain oxygen atoms of *Ala 305* and *308*, thus further fixing the N-terminus and increasing the dimerization formation (Fig. 2b).

In region **II (**Arg277-Phe286, located in the loop between α1 and α2), a total of six hydrogen bonds were found between A and B molecules (Fig. 2c; only the loop from molecule A is shown here for simplicity). The side-chain of Arg277 of subunit A forms a strong hydrogen bond with the main-chain oxygen of *Gly316* from protomer B (Fig. 2c). The main-chains of Gly278 and Glu280 interact with the side-chain of *Arg319* with two hydrogen bonds. Asn285 with its main-chain and side-chain makes three hydrogen bonds with the main-chains of *Ser318* and *Ile320.* Arg277, Gly278, Glu280, as well as Asn285 form a hydrogen-bond network with the adjacent protomer, thus reducing the flexibility of the longest loop between α1 and α2 of the N-CTD. This implies that a single N-CTD does not possess an ideally stable conformation. Furthermore, residues Arg277 and Asn285 are absolutely conserved in the *alpha-* (i.e. HCoV-NL63), *beta-* (i.e. SARS-CoV-2, SARS-CoV, and MERS-CoV), and *gamma* (i.e. IBV)*-*CoVs (Fig. 4), suggesting that these hydrogen bonds in the dimerization are conserved in various CoVs.

In region **IV (**Gly328-Leu339, β2 strand region), the β2 of monomer A interacts with β2 strand from subunit B to make an intermolecular antiparallel β-sheet, with a total of 10 main-chain hydrogen bonds formed by five pairs Thr329-*Leu339*, Leu331-*Ile337*, Tyr333-*Gly335*, Gly335-*Tyr333*, and Ile337-*Leu331* (Fig. 2d), leading to a significant increase in the stability of the N-CTD homo-dimer. This observation is in good agreement with previous results [29, 30, 33]. In the β2 strand region, Thr334 of SARS-CoV-2 N-CTD is replaced by His335 in SARS-CoV (Figs. 2d and 4). The His335Ala mutation of SARS-CoV N-CTD reduces its RNA binding affinity by about 50% [30]. Takeda *et al*. explained that the β-sheet region could be part of the N-CTD nucleotide-binding site [30]. His335 in SARS-CoV is unique compared to the corresponding residue in other CoVs (Fig. 4, indicated by a red arrow). It will be worth investigating the effect of Thr instead of His in SARS-CoV-2 N-CTD on its RNA binding affinity in the future.

In the left two regions, **III** and **V**, the hydrophobic interactions between the two subunits are dominant. In region **III** (Ile304-Met322), residues Ile304, Ala305, Ala308, Pro309, Phe314, and Phe315 from subunits A and B interact with each other to form a strong hydrophobic core (Fig. 2e). In region **V**, residues Phe346, Val350, Leu353 and Ile 357 of the α5 helix in protomer A display hydrophobic interactions with *Thr329* (Cβ and Cγ atoms of the side-chain), *Met322*, *Leu331* and *Ile320* of subunit B, respectively (Fig. 2f). Among all the residues mentioned here, only Pro309 is absolutely conserved in the *alpha-*, *beta-*, and *gamma-*CoVs; however, the hydrophobic property of the left residues is almost retained in different CoV genera (Fig. 4), indicating that the hydrophobic cores in regions **III** and **V** are preserved for N protein dimerization.

With the hydrogen bonds and hydrophobic interactions mentioned above, a very stable N-CTD dimer is formed. This dimer was confirmed in solution by gel filtration assay (Fig. 2g; the sample used for this assay is shown in SDS-PAGE). In addition, a size exclusion chromatography coupled to multi-angle light scattering (SEC-MALS) assay was performed in order to directly detect the molecular mass (M. M.) of the SARS-CoV-2 N-CTD in solution (supplementary Fig. S1). According to this assay, the M. M. is 26.8 ± 0.7 kD and closely matches with the theoretical value (N-CTD dimer: ~ 26.7 kD, Fig. S1). This intertwined dimer is very likely a functional unit of the SARS-CoV-2 N protein.

The crystal of the SARS-CoV N-CTD consists of an octamer formed by four homo-dimers per ASU (PDB code: 2CJR, [29]). A putative helical oligomer CTDs-RNA model was proposed by packing these octamers [11, 29, 30]. In our case, four molecules formed by two dimers exist in an ASU. The dimeric packing pattern is different from that of SARS-CoV N-CTD [29]. The interface area of the SARS-CoV-2 two dimers is about 220 Å^2^ (Chain A and Chain C) as determined by the PDBePISA server [42]. The complex (dimer-dimer) formation significance score (scale from 0 to 1) is 0 compared to 0.971 for the intra-dimer (monomer-monomer) formation. In addition, we did not detect tetramers or higher oligomers in the gel filtration assay. Therefore, the tetramer per ASU in our crystal is likely to result from crystal packing.

On the other hand, self-association (dimerization or oligomerization) of CoV N plays a central role during virus replication [15]. The C-terminal tail of the HCoV-229E N protein is important for its oligomerization [44]. Using the C-terminal tail peptide could interrupt the self-association of the N-CTD of HCoV-229E to further attenuate virus replication [44]. Therefore, designing the proper peptides to interfere with the dimerization or oligomerization of the N protein is a potential anti-viral strategy to counter the SARS-CoV-2.

### Comparison of the N-CTD structures in various coronaviruses

**Fig. 3:**
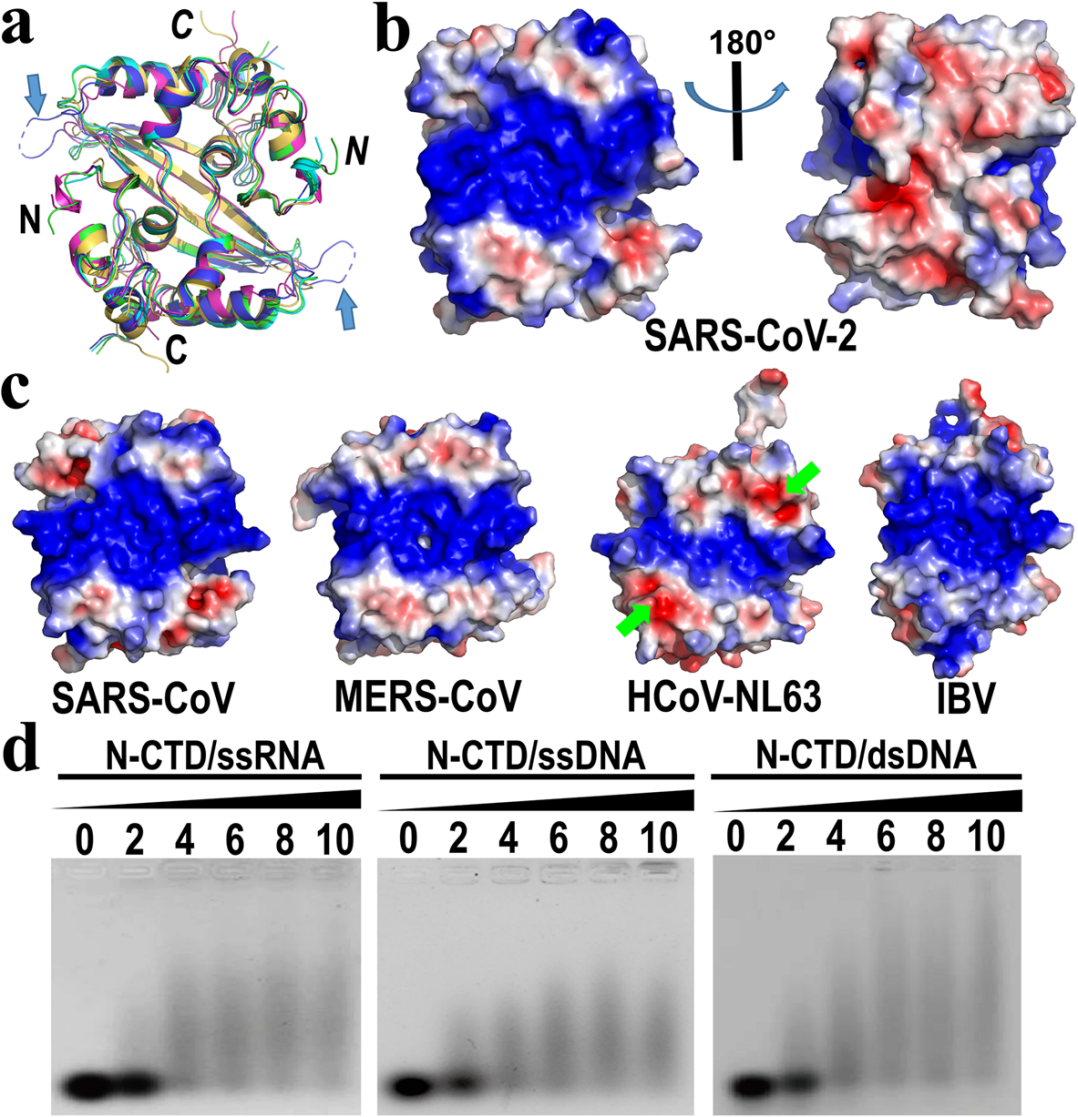
Structural comparison of N-CTDs from various CoVs and oligonucleotide-binding assay of the SARS-CoV-2 N-CTD. **a** Superposition of N-CTDs from SARS-CoV-2 (cyan), SARS-CoV (green, PDB code: 2CJR), MERS-CoV (blue, PDB code: 6G13), HCoV-NL63 (purple, PDB code: 5EPW) and IBV (yellow, PDB code: 2GE8). The N- and C- termini of subunits A and B (in italics) are labelled. The long insertion region of each β-hairpin in the MERS-CoV N-CTD is marked by a blue arrow. **b** The electrostatic surface of the SARS-CoV-2 N-CTD dimer structure. The contouring level is -3 kBT/e (red) to 3 kBT/e (blue). The helices side of the rectangular slab dimer shows a strong positively charged region for the potential RNA binding (left image). The opposite β-sheet side is almost a negatively charged and neutral region (right image). The orientation of the left image is same as in the ribbon representations in (**a**). **c** The electrostatic surfaces of the N-CTD dimers from SARS-CoV, MERS-CoV, HCoV-NL63 and IBV. The PDB codes used here are same as in (**a**). Residues Glu243/244 of each subunit in HCoV-NL63 are marked by green arrows. All orientations are same as in the ribbon view in (**a**). All figures were generated using the program PyMOL (https://pymol.org). **d** The EMSA experiment of the SARS-CoV-2 N-CTD. The mobility shifts of ssRNA, ssDNA and dsDNA bound to the N-CTD are shown, respectively. The molar ratios of N-CTD/nucleotides in each lane are indicated

A structural similarity search of SARS-CoV-2 N-CTD subunit A against the Protein Data Bank was performed by the DaLi server [40]. The results indicate that the three-dimensional structures of N-CTDs are very conserved in *alpha*-, *beta*-, and *gamma*-CoVs, despite the lowest sequence identity of 27% between SARS-CoV-2 and IBV (Table 2 and Fig. 3a).

**Table 2:** Structural comparisons of the SARS-CoV-2 N-CTD with other homology proteins

SARS-CoV-2 N-CTD						
N-CTDs	PDB/Chain	Z-score	RMSD (Å)	Cα^a^	% id^b^	References
SARS-CoV	2CJR/C	19.1	0.4	109/113	95	[29]
MERS-CoV	6G13/D	18	1.1	109/114	54	[37]
HCoV-NL63	5EPW/B	13.8	2.1	102/110	32	[36]
IBV	2GE8/D	13.2	1.8	106/111	27	[34]

^a^ aligned Cα atoms/total Cα atoms; ^b^ Sequence identity

All values were calculated by the DaLi server [40]

**Fig. 4:**
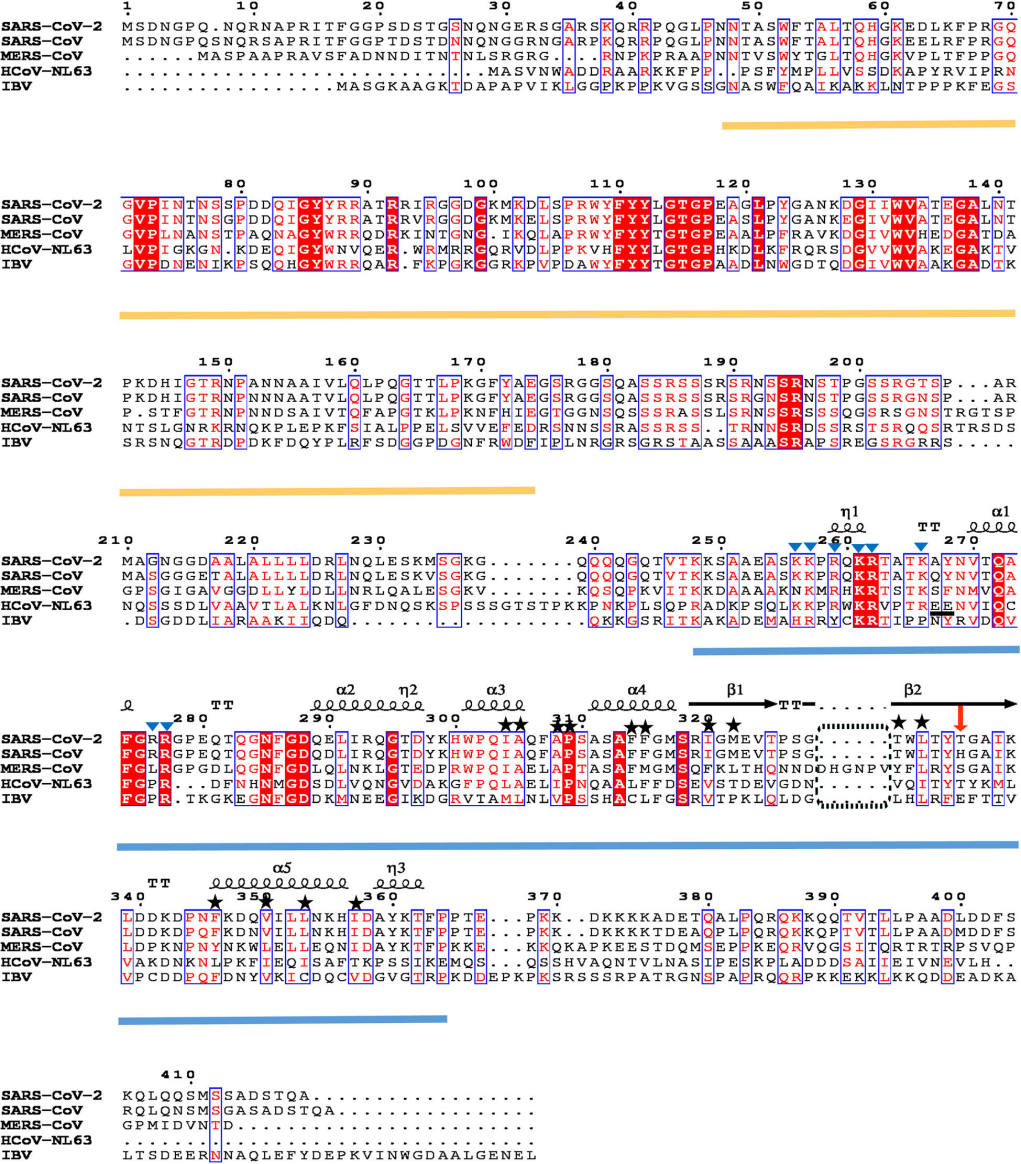
Multiple sequence alignment of N proteins among different CoVs. The sequence accession numbers are: SARS-CoV-2, GenBank: MN908947; SARS-CoV, GenBank: AY274119; MERS-CoV GenBank: KC164505; HCoV-NL63, GenBank: AY567487; and IBV, GenBank: M95169. SARS-CoV-2, SARS-CoV and MERS-CoV belong to *beta*-CoV genus. HCoV-NL63 is from *alpha*-CoV group while IBV is from *gamma*-CoV group. The N-terminal domain (NTD) and C-terminal domain (CTD) of the N protein are marked by orange and blue lines, respectively. The secondary structures of the SARS-CoV-2 N-CTD are indicated. The long insertion of the β-hairpin in the MERS-CoV N-CTD is indicated by a dashed black box. The residues involved in forming two hydrophobic cores for the N-CTD dimerization are labelled with the black stars. The basic residues referring to construct the positive charge groove are indicated by blue triangles. Two acidic residues Glu243/244 of HCoV-NL63 are underlined by a short black line. The non-conserved Thr334 between SARS-CoV-2 and SARS-CoV is indicated by a red arrow. This figure was generated by the program ESPript [43]

The structure most similar to SARS-CoV-2 N-CTD is that of SARS-CoV N-CTD (95% sequence identity; PDB code: 2CJR, [29]) with an RMSD 0.4 Å over 109 Cα atoms out of 113 residues. The RMSD values between the SARS-CoV-2 N-CTD and the corresponding domains in MERS-CoV [37], HCoV-NL63 [36], as well as IBV [34] are 1.1 Å (PDB code: 6G13), 2.1 Å (PDB code: 5EPW), and 1.8 Å (PDB code: 2GE8), respectively (Table 2). In addition to the differences of the flexible N- and C-termini among these N-CTDs, the loop between β1 and β2 of MERS-CoV is obviously longer than those from other CoVs (Figs. 3a and 4). It remains to be elucidated whether the long insertion into the β-hairpin plays any role in viral replication.

SARS-CoV N-CTD is capable of interacting with ssRNA, ssDNA and dsDNA in vitro without nucleotide sequence specificity [29, 30], suggesting the N-CTD nucleotides binding is most likely due to nonspecific charge interactions. Residues 248–280 in the SARS-CoV N-CTD within a large number of Lys/Arg are essential for RNA binding [29, 30]. The corresponding residues, 247-T**KK**SAAEAS**KK**P**R**Q**KR**TAT**K**AYNVTQAFG**RR**GP-279, are conserved in SARS-CoV-2, with only one amino acid Ala267 (underlined) replacement to Gln268 in SARS-CoV (Fig. 4).

The electrostatic surface of the SARS-CoV-2 N-CTD in our structure displays a significantly positively charged region located at the helical side of the dimer (Fig. 3b, left), indicating a potential RNA-binding site. Further analysis revealed the clustering of positive charges due to the N-termini eight basic residues (Lys256, Lys257, Arg259, Lys261, Arg262, Lys266, Arg276 and Arg277) of each subunit (Lys248/249 are invisible in our structure because of the lack of electron density, as mentioned above). On the other hand, the β-sheet side of dimer shows an almost acidic and neutral environment (Fig. 3b, right), suggesting an unideal RNA binding site. Although this β-hairpin site related to RNA binding in vitro was reported in SARS-CoV [30], we need to further confirm whether a similar binding site exists in SARS-CoV-2 or not.

Next, we checked the electrostatic surface of the homology N-CTDs from *alpha*-, *beta*-, and *gamma*-CoVs (Fig. 3c). Most of the eight positive residues (see above) were conserved in SARS-CoV, MERS-CoV, HCoV-NL63, and IBV (Fig. 4). A similar surface with the clustering of positive charges exists in all N-CTDs, suggesting a conserved viral RNA binding pattern throughout the evolution of various CoVs. However, we noticed that the positively charged groove in HCoV-NL63 is much smaller than those of other CoVs. We found two acidic residues Glu243/244 are unique in HCoV-NL63, which could significantly reduce the positive charge around this region (Fig. 3c). These two residues are replaced by neutral and/or hydrophobic residues in other CoVs (Fig. 4). How the different distribution of the electronic surface affecting the viral RNA binding affinity in various CoVs is interesting to be investigated later.

### Nucleotides-binding activity of the SARS-CoV-2 N-CTD

We have analyzed the potential RNA/DNA binding ability of the SARS-CoV-2 N-CTD (see above), however, the direct evidence for the nucleotide-binding activity of this N-CTD is scarce. Therefore, we performed the electrophoretic mobility shift assay (EMSA) (Fig. 3d). The 17-mer ssRNA oligonucleotides 5′-UGUUCUCUAAACGAACU-3′, which are located in the 5′ untranslated region of the SARS-CoV-2 genome (GenBank: MN908947, nucleotides 60–76), were used in our assay. This 17-mer RNA also exists in the SARS-CoV genome and is referred to as the stem-loop 3 (SL3, including the transcriptional regulatory sequences (TRS)) [45]. The amount of the free 17-mer RNA decreased with the addition of more N-CTD protein (Fig. 3d, right), indicating the binding affinity between RNA and the proteins. Furthermore, the N-CTD could interact with this RNA’s mimic DNA as well as dsDNA nucleotides (Fig. 3d, middle and left). Taken together, the SARS-CoV-2 N-CTD displays the binding activity of both RNA and DNA, which is similar to that of the SARS-CoV N-CTD [29, 30].

## Conclusions

CoV nucleocapsid protein, as an essential multifunctional viral protein, plays roles in the viral RNA packaging and the regulation of host cellular processes, such as cell cycle and innate immune response. In an effort to better understand the structure and functions of the SARS-CoV-2 N protein, our study provides the structural data of its C-terminal domain and reports the nucleotide-binding activity of the corresponding region. In addition, CoV N protein is involved in binding various viral and host proteins. Despite of all recent discoveries, the structure of N protein in complex with viral RNA or its partner proteins is still missing. Future research should focus on demonstrating such structure(s).

## Materials and methods

### Recombinant production of the C-terminal domain of SARS-CoV-2 nucleocapsid protein (N-CTD)

The cDNA plasmids (WH-nCoV in pBluescript II SK (+)) encoding for the full-length SARS-CoV-2 nucleocapsid protein were purchased from General Biosystems, Anhui, China. This cDNA sequence is identical to the sequence of the *N* gene in SARS-CoV-2 isolated from Wuhan-hu-1 (GenBank: MN908947, [2]). The SARS-CoV-2 N-CTD contains the residues Lys248-Pro364 of the N protein. The corresponding *N-CTD* gene was amplified by PCR with the forward primer 5′-CTAGCTAGCAAGAAATCTGCTGCTGAGGCTTC-3′ and the reverse primer 5′-CCGCTCGAGTTATGGGAATGTTTTGTATGCGTC-3′. The PCR product was further digested using the *NheI* and *XhoI* restriction endonucleases and ligated into modified pET28a plasmids containing an N-terminal hexa-histidine tag and a Tobacco Etch Virus (TEV) protease cleavage site (the modified plasmids were kindly provided by our colleague Prof. Qiang Chen). The recombinant plasmid DNA was verified by sequencing (Youkang Biology Company, Chengdu, China).

The corrected N-CTD pET28a plasmid was transformed into *E. coli* BL21(DE3) (Novagen). The transformed cells were grown overnight at 37 °C in 50 mL Luria-Broth (LB) medium supplemented with kanamycin at a final concentration 50 μg/mL. The culture was then inoculated into 2 × 1 L LB medium the following day. When the OD600 value of the culture reached 0.6–0.8, overexpression of the *N-CTD* was induced for 16 h through 0.5 mM isopropyl-D-thiogalactoside (IPTG) supplementation at 18 °C. The 2 x lL culture was subsequently harvested by centrifugation for 15 min, 4000 rpm at 4 °C. Pellets were re-suspended in 50 mL buffer A (20 mM Tris-HCl, 10 mM imidazole, 500 mM NaCl, pH 7.5) and lysed by sonication on ice. Debris was removed by centrifugation for 30 min at 20,000 rpm at 4 °C. The supernatant was applied to the Ni Sepharose™ Fast Flow beads (GE Healthcare). The His-tagged N-CTD protein was eluted using buffer B (20 mM Tris-HCl, 500 mM imidazole, 500 mM NaCl, pH 7.5) with a step-gradient method. The target protein was then cleaved by TEV protease (leaving three extra residues Gly-Ala-Ser at the N-terminus of the N-CTD) and dialyzed against buffer C (20 mM Tris-HCl, 150 mM NaCl, pH 7.5) overnight at 4 °C. Next day, the target protein was applied to Nickel beads again to remove the uncleaved His-tag protein. The N-CTD without the His-tag protein was further purified by gel filtration (Superdex 200 Increase 10/300 GL, GE Healthcare) using buffer C. The quality of the N-CTD protein was checked by sodium dodecyl sulfate polyacrylamide gel electrophoresis (SDS-PAGE, Fig. 2g).

### Crystallization and data collection

Purified SARS-CoV-2 N-CTD was concentrated to ~ 40 mg/mL in buffer C. Crystallization experiments were performed at 291 K by the sitting-drop vapor-diffusion method with 1 μL protein plus 1 μL reservoir. The commercial screen kits Index, SaltRx 1/2, Crystal Screen 1/2, PEG/Ion Screen 1/2, PEGRx 1/2 (Hampton Research) as well as Structure screen 1/2, PACT premier and JCSG plus (Molecular Dimensions) were used. Crystals were observed under Index condition No.57 (0.05 M ammonium sulfate, 0.05 M Bis-Tris pH 6.5, 30% v/v pentaerythritol ethoxylate (15/4 EO/OH) and Structure screen 1 No. 2 (0.2 M ammonium acetate, 0.1 M sodium acetate pH 4.6, 30% PEG4000). Crystals were reproduced under the Index No.57 condition within 3 ~ 5 days. Using 25% PEG400 as a cryo-protected agent, the optimized crystals were shock-cooled in liquid nitrogen. A diffraction dataset to 2.0 Å was collected with the X-ray wavelength 0.97851 Å at Shanghai synchrotron radiation facility (SSRF) beamline BL19U1, Shanghai, China. This dataset was processed by *XDS* [46] and scaled with *Aimless* in *CCP4* [47]. The space group is *P*_*1*_, with unit-cell parameters *a* = 43.76 Å, *b* = 49.46 Å, *c* = 68.82 Å, α = 106.79°, β = 90.04°, γ = 97.79°. See Table 1 for the diffraction data statistics.

### Structure determination and refinement

The structure of N-CTD was solved by the molecular replacement method with the program *Phenix. Phaser* [48] using the modified search model (PDB ID: 2CJR, Chain A, [29]). Four molecules (Chain A, B, C and D) within the asymmetric unit (ASU) were identified. The initial model of the N-CTD was circularly rebuilt and refined using programs *Coot* [49] and *Refmac5* [50]. Residues 256–364 of Chain A, 252–364 of Chain B, 253–364 of Chain C and 257–364 of Chain D were successfully built in the final structure model with *R*_*factor*_ and *R*_*free*_ of 0.189 and 0.238, respectively. The final model refinement statistics are listed in Table 1.

### SEC-MALS assay

In order to directly measure the molecular mass of the SARS-CoV-2 N-CTD in solution, the size exclusion chromatography coupled to multi-angle light scattering (SEC-MALS) assay was performed (supplementary Fig. S1). The purified 50 μL N-CTD (~ 2 mg/mL) proteins (totally 100 μg) were injected to the column Superdex 200 Increase 10/300 GL (GE Healthcare) to run with the flowrate at 0.5 ml/min. The light scattering signals and the refractive index profiles were collected with the miniDAWN and Optilab (Wyatt Technology). The molecular mass was calculated by the software ASTRA (Wyatt Technology).

### Electrophoretic mobility shift assay (EMSA)

The method of this assay was modified according to previous literature [29, 30]. The 17-mer ssRNA oligonucleotides 5′-UGUUCUCUAAACGAACU-3, which are located in the 5′ untranslated region of the SARS-CoV-2 genome (GenBank: MN908947, nucleotides 60–76), was purchased from Youkang Biology Company (Chengdu, China). In addition, the corresponding DNA (5′-TGTTCTCTAAACGAACT-3′) and its complement oligonucleotides (5′-AGTTCGTTTAGAGAACA-3′, for double stranded DNA formation) were purchased from Sangon Biotech Co., Ltd. (Shanghai, China). The dsDNA was further prepared by equal numbers of the ssDNA and its complement DNA after denaturing at 95 °C and renaturing at room temperature. Next, the 1, 2, 3, 4 and 5 μL of the N-CTD (stock concentration: 100 μM) were incubated with 5 μL of dsRNA, ssDNA as well as dsDNA (each stock concentration: 10 μM) at 4 °C for 30 min, respectively. The N-CTD/nucleotides molar ratios are 2:1, 4:1, 6:1, 8:1 and 10:1. The sample of oligonucleotides without protein was used as the control. Reaction in each tube was set up to 10 μL aliquots totally. All the experiments were performed in the buffer: 20 mM HEPES, 150 mM NaCl, pH 7.5. These reaction samples were then separated on 1% agarose gel on ice at 60 V for 1 h. The results were visualized by Tanon-3500B imager (Tanon Co., Ltd., Shanghai, China).

## Supplementary information

**Additional file 1: Fig. S1.** SEC-MALS assay of the SARS-CoV-2 N-CTD. The size exclusion chromatography coupled to multi-angle light scattering (SECMALS) assay was performed to determine the molecular mass (M. M) of the SARSCoV-2 N-CTD in solution. The M. M is 26.8 ± 0.7 kD (indicated by the red arrow; the theoretical N-CTD dimer is ~ 26.7 kD). LS: light scattering; dRI: differential refractive index.

## Data Availability

PDB code: 7C22.
