# Supplementary material for: Structural characterization of the C-terminal domain of SARS-CoV-2 nucleocapsid protein
Source: Mol Biomed. 2020 Aug 6;1:2. doi: 10.1186/s43556-020-00001-4 (PMC7406681; doi:10.1186/s43556-020-00001-4)
Supplement: Supplementary file 1 — Additional file 1: Fig. S1. SEC-MALS assay of the SARS-CoV-2 N-CTD. The size exclusion chromatography coupled to multi-angle light scattering (SECMALS) assay was performed to determine the molecular mass (M. M) of the SARSCoV-2 N-CTD in solution. The M. M is 26.8 ± 0.7 kD (indicated by the red arrow; the theoretical N-CTD dimer is ~ 26.7 kD). LS: light scattering; dRI: differential refractive index. [file 43556_2020_1_MOESM1_ESM.pdf]

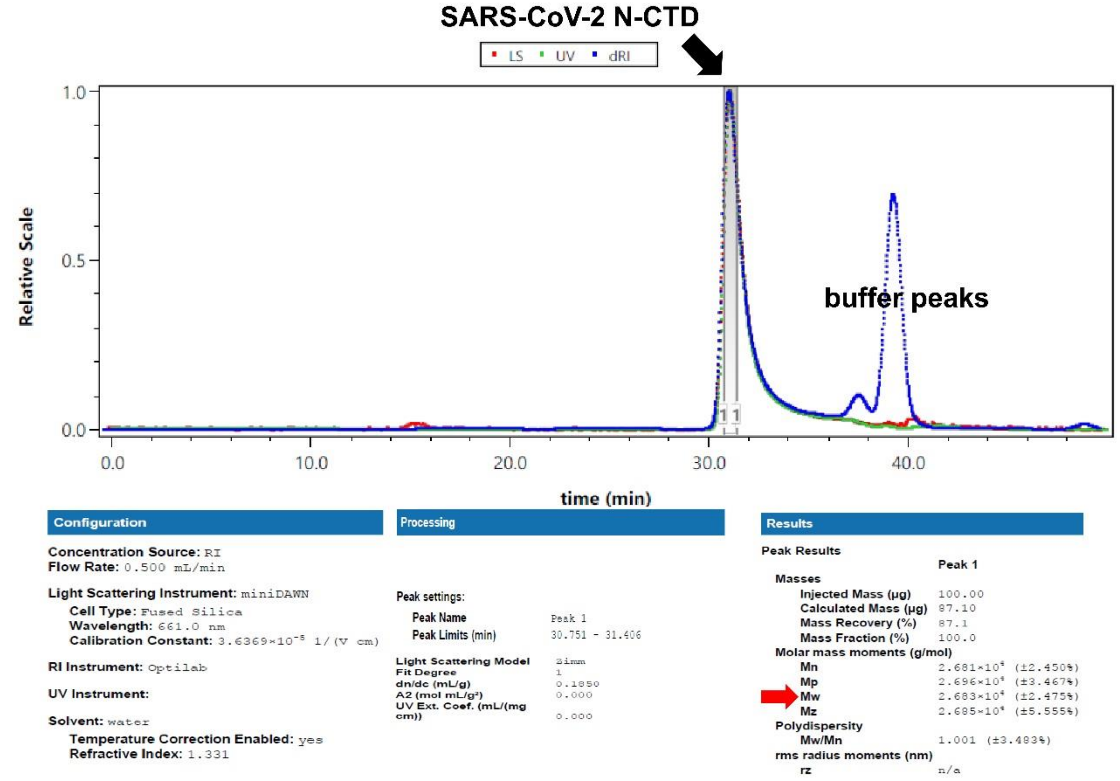

Fig. S1. SEC-MALS assay of the SARS-CoV-2 N-CTD.

The size exclusion chromatography coupled to multi-angle light scattering (SEC-MALS) assay was performed to determine the molecular mass (M. M) of the SARS-CoV-2 N-CTD in solution. The M. M is 26.8 ± 0.7 kD (indicated by the red arrow; the theoretical N-CTD dimer is ~26.7 kD). LS: light scattering; dRI: differential refractive index.
